# Supplementary material for: Transdifferentiation of plasmatocytes to crystal cells in the lymph gland of Drosophila melanogaster
Source: EMBO Rep. 2025 Mar 12;26(8):2077–97. doi: 10.1038/s44319-025-00366-z (PMC12019564; doi:10.1038/s44319-025-00366-z)
Supplement: Supplementary file 1 — Movie EV1 [file 44319_2025_366_MOESM1_ESM.zip › video and legend/movie legend.rtf]

MOVIE EV1 – Long term live imaging of cultured lymph glandsA single lobe, from a cultured lymph gland was cultured and imaged for 12h. Single positive Eater-dsRed cells (plasmatocytes) were observed to start to co-express BcF6-GFP (crystal cell marker). At around 9h of culture, “dark spots” were observed within the BcF6-GFP expression domain of a double-positive cell. Scale bar represents 20μ.
