# Supplementary figures and images for: Transdifferentiation of plasmatocytes to crystal cells in the lymph gland of Drosophila melanogaster
Source: EMBO Rep. 2025 Mar 12;26(8):2077–97. doi: 10.1038/s44319-025-00366-z (PMC12019564; doi:10.1038/s44319-025-00366-z)

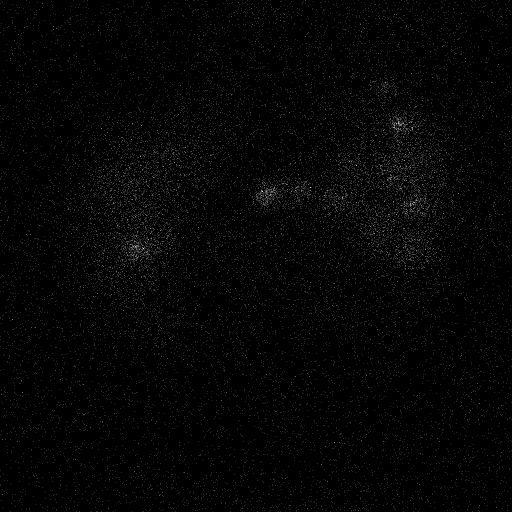

Supplement: Supplementary file 3 — Source data Fig. 2 [file 44319_2025_366_MOESM3_ESM.zip › Figure 2/2B/SuHLacZ_BcGFP_Anti-Nimrod.tif]

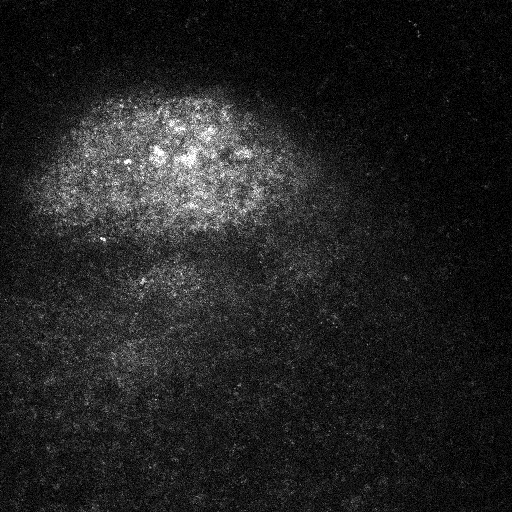

Supplement: Supplementary file 4 — Source data Fig. 3 [file 44319_2025_366_MOESM4_ESM.zip › Figure 3/3B/Lz-Gal4_lineage_tracing_Anti-Nimrod.tif]

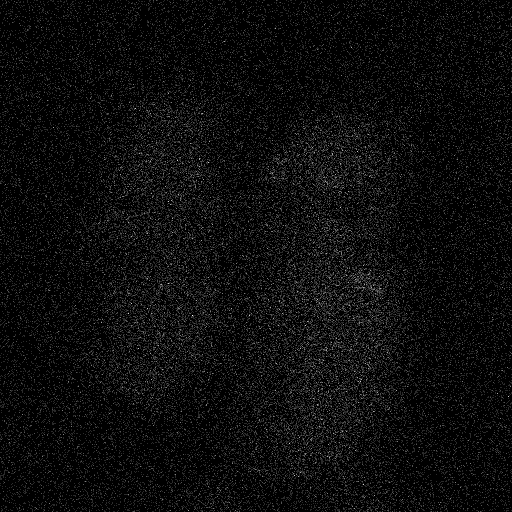

Supplement: Supplementary file 5 — Source data Fig. 4 [file 44319_2025_366_MOESM5_ESM.zip › Figure 4/LG_phagocytosis assay_BcGFP_Latex_Nimrod.tif]

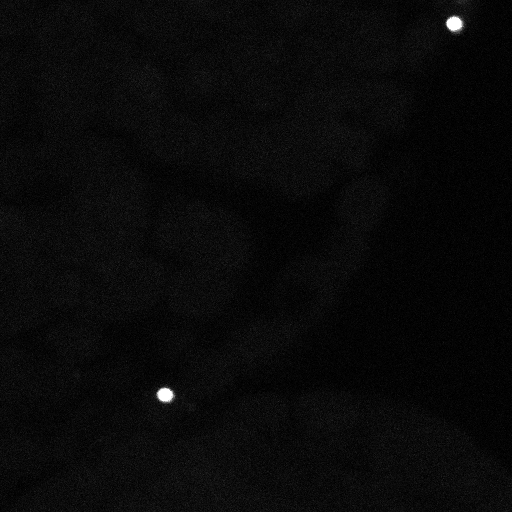

Supplement: Supplementary file 5 — Source data Fig. 4 [file 44319_2025_366_MOESM5_ESM.zip › Figure 4/LG_phagocytosis assay_BcGFP_Latex_Nimrod_Zoom.tif]
