# Supplementary material for: Transdifferentiation of plasmatocytes to crystal cells in the lymph gland of Drosophila melanogaster
Source: EMBO Rep. 2025 Mar 12;26(8):2077–97. doi: 10.1038/s44319-025-00366-z (PMC12019564; doi:10.1038/s44319-025-00366-z)
Supplement: Supplementary file 8 — Expanded View Figures [file 44319_2025_366_MOESM8_ESM.pdf]

Expanded View Figures

Figure EV1. Notch activity in cortical plasmatocytes and co-localization of P1 with crystal cell markers.

(**A**) Hml-CytoplasmicDsRed was used to mark mature haemocytes of the cortical region and NRE-eGFP as a reporter of Notch activation. NRE-eGFP expression is restricted to the cortical region of the lymph gland showing that Notch activation is restricted to mature and maturing plasmatocytes. Beneath the main image is a XZ slice of the same lymph gland illustrating the specificity of the two markers to the cortical region. Dotted lines define the contour of the primary lymph gland lobes. (**a′**) Shows an orthogonal section of the Lymph glands shown in (**A**). The horizontal dashed line in (**A**) represents the location of the slice. (**B**) Bc-GFP lymph glands stained with anti-Nimrod (red). Bc-GFP is a marker of crystal cells that co-localizes in a subset of cortical cells with the plasmatocyte marker Nimrod (P1) (white arrowheads). Dotted lines define the contour of the primary lymph gland lobes. (**b′**) 10-fold magnification of the inset in (**B**). (**C**) Lz-Gal4, UAS-GFP lymph glands stained with P1 (anti-Nimrod) (red). In agreement with the staining in (**A**), double-positive cells can be observed in the cortical region of the lymph gland (white arrowheads). Dotted lines define the contour of the primary lymph gland lobes. (**c′**) 10-fold magnification of the inset in (**C**). Scale bars represent 100 µm in (**A–C**), and 10 µm in (**a′–c′**).

© The Author(s)

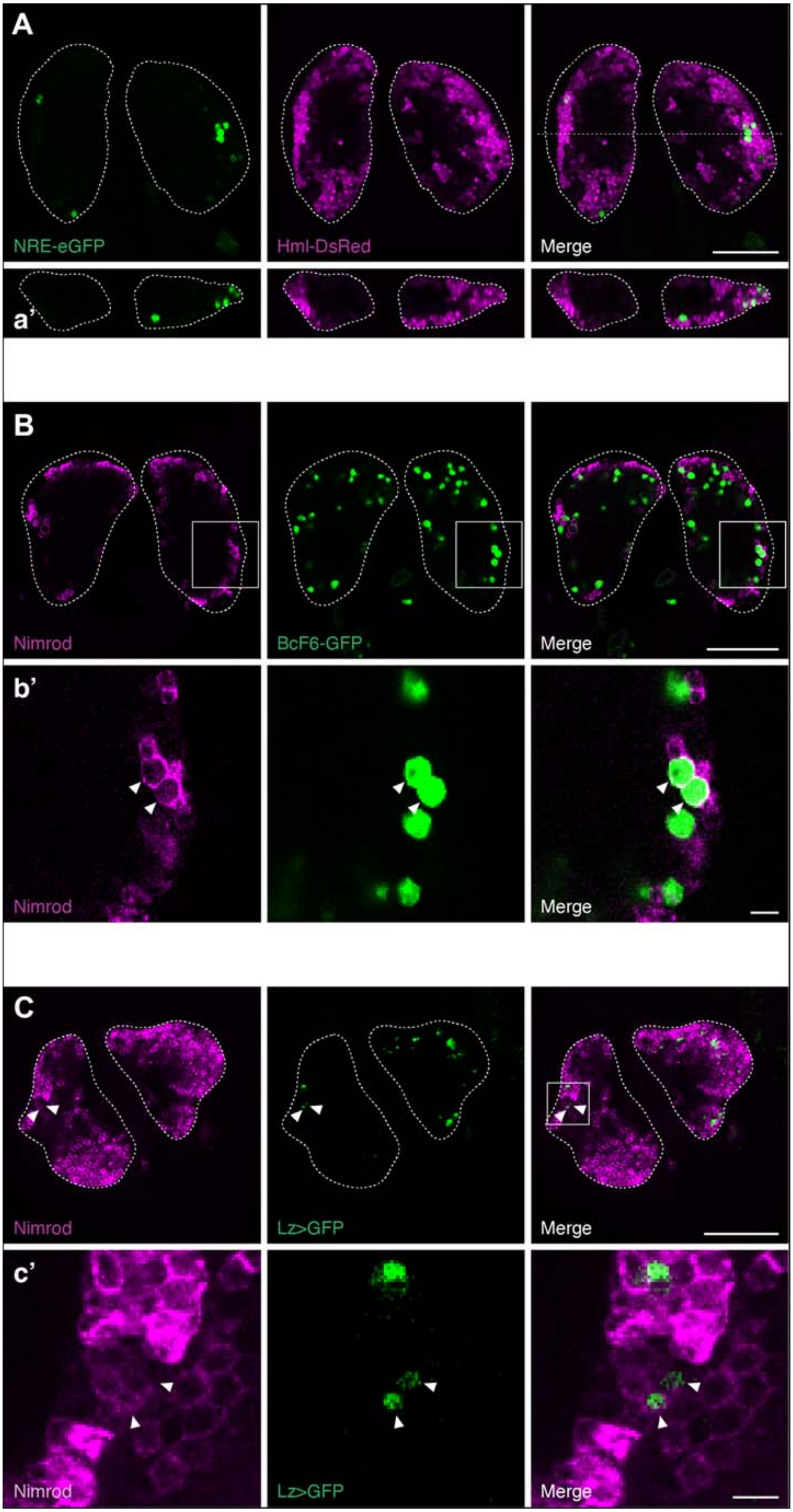
A
NRE-eGFP
Hml-DsRed
Merge
a'
B
Nimrod
BcF6-GFP
Merge
b'
Nimrod
Merge
C
Nimrod
Lz>GFP
Merge
c'
Nimrod
Lz>GFP
Merge

© The Author(s)

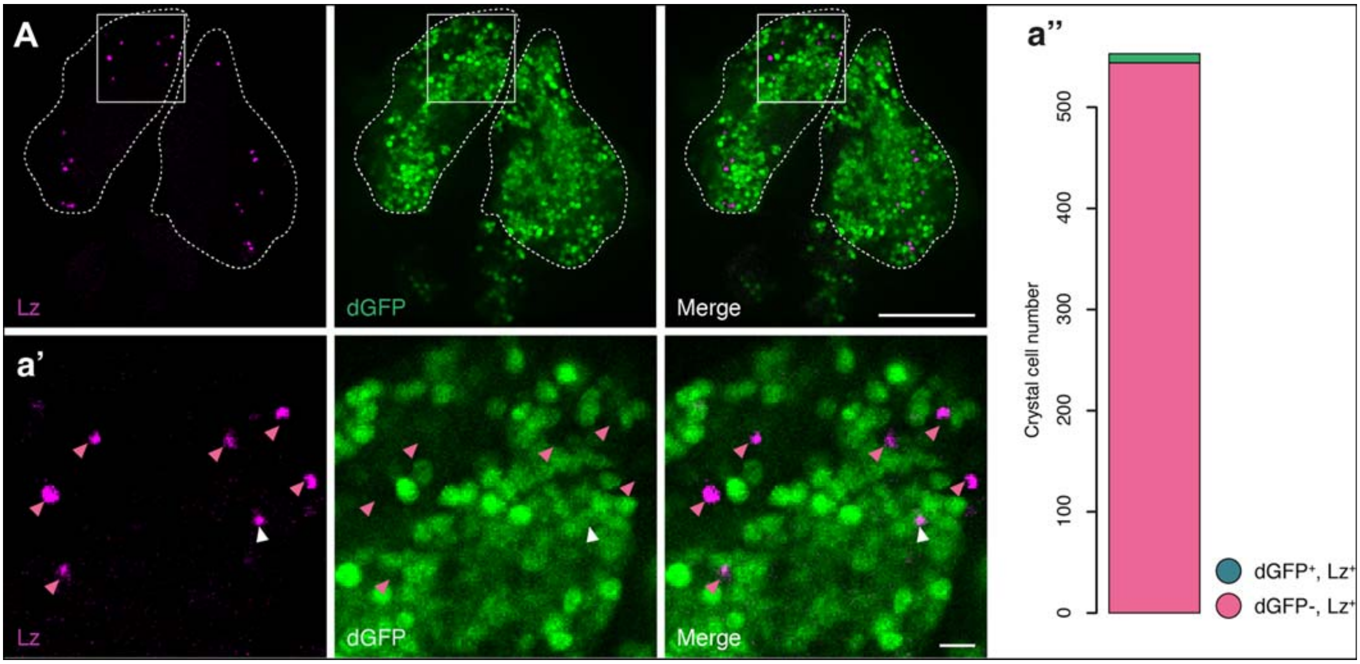

Figure EV2. Eater-Gal4 expression is rapidly lost in Crystal cells.

Eater-Gal4 driving destabilized GFP expression is lost rapidly in crystal cells as shown by the lack of co-localization with Lozenge. Dotted lines define the contour of the primary lymph gland lobes. (**a**′) 10-fold magnification of the inset in (**A**). Magenta arrows mark cells positive for Lz alone. Rare double-positive cells are marked by white arrows. (**a**′′) Scale bar represents 100 μm in (**A**) and 10 μm in (**a**′).

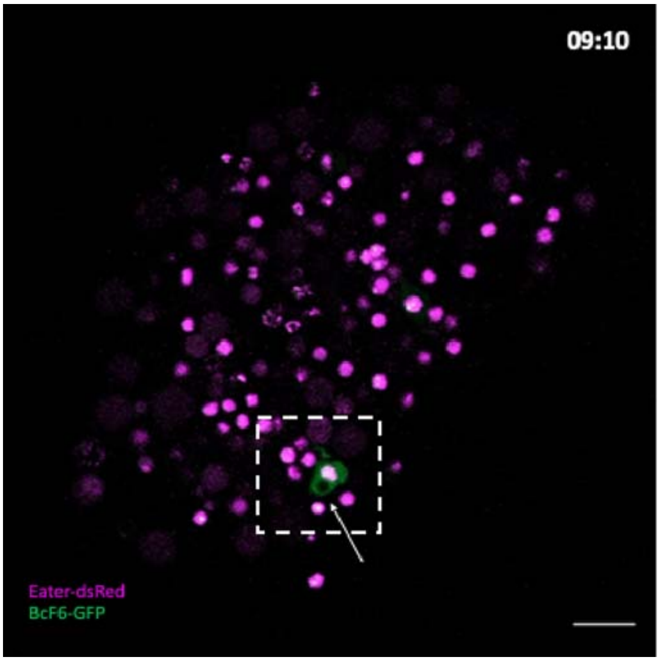

Figure EV3. "Dark spots" observed in double-positive cells from cultured lymph glands.

Image extracted from timelapse represented in Movie EV1. At around 9 h of culture, "dark spots" were observed within the BcF6-GFP expression domain of a double-positive cell (inside white dotted box), suggesting PPO1 production by transdifferentiating cells. Scale bar represents 20 μm.

 © The Author(s)

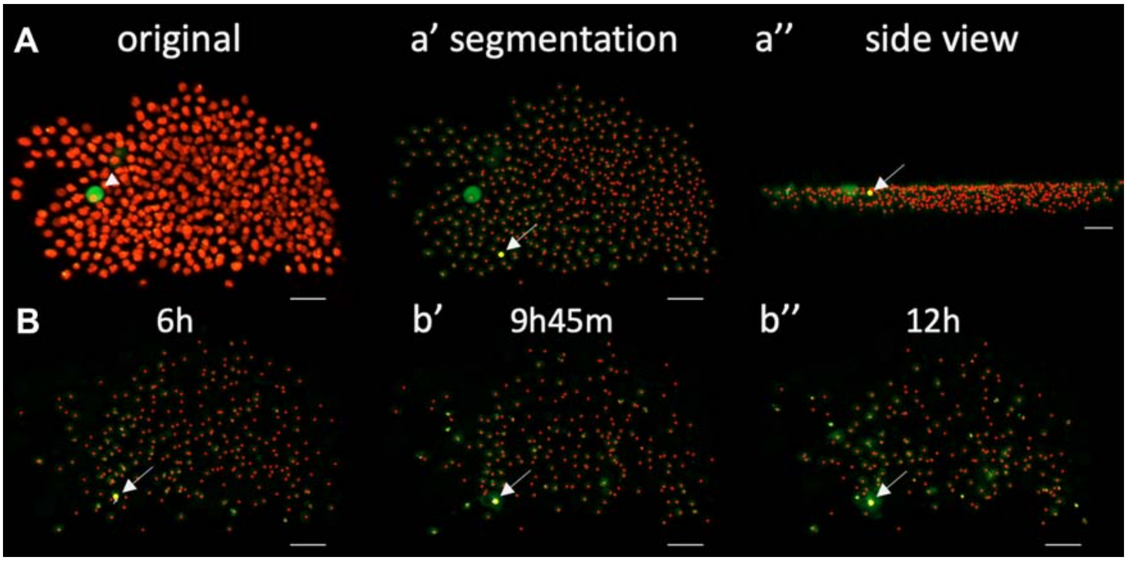

Figure EV4. Transdifferentiation of plasmatocytes into crystal cells can occur in the cortical zone of the lymph gland.

(**A**) Lymph gland from Movie EV1 with *Eater*-DsRed and BcF6-GFP markers. Crystal cells can be observed with cytoplasmatic GFP signal (green arrowhead). (**a′**) Spot segmentation of DsRed nuclei allows the tracking of individual cells throughout the experiment. Two *Eater*-DsRed$^+$ BcF6-GFP$^-$ cells are highlighted with a white spot and arrow. Both these nuclei are in the second row of nuclei from the surface of the lymph gland. (**a″**) At 12 h the cytoplasm around these nuclei is GFP$^+$. (**B**) An independent sample of lymph gland ex vivo culture, with examples of BcF6-GFP$^+$ marked with green arrows. (**b′**) At 0 h, it is possible to identify 4 *Eater*-DsRed$^+$ BcF6-GFP$^-$ cells that will gain GFP expression. Two of these cells are in the second row of nuclei (arrows) and the other two are deeper in the tissue (white arrowheads). (**b″**) At 12 h the cytoplasm around these nuclei is GFP$^+$. Scale bars represent 15 µm (**A**, **a**′, **a**″) and 10 µm (**B**, **B**′, **B**″).

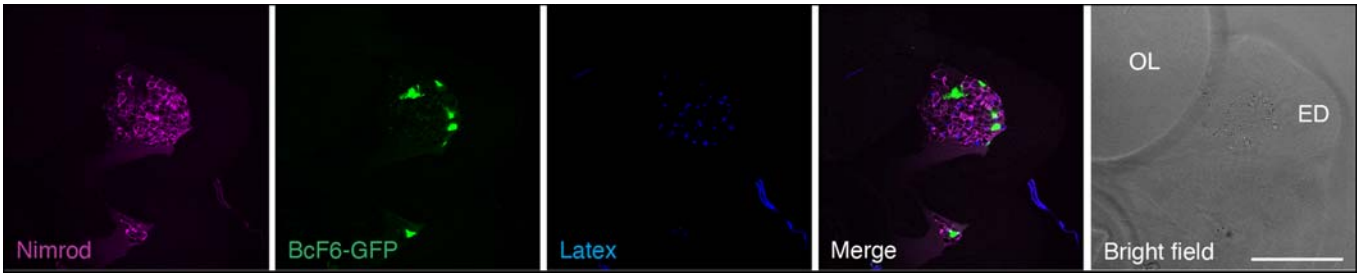

Figure EV5. Latex beads are taken up exclusively by haemocytes.

Latex beads co-localize only with the haemocyte patch (outlined in white) identified by the expression of Bc (crystal cells) or P1 (plasmatocytes). Cells of the neighboring eye disc (ED) and optic lobe (OL), visible in the merge image, show no phagocytosis of the latex beads. Scale bar represents 100 μm.

 © The Author(s)

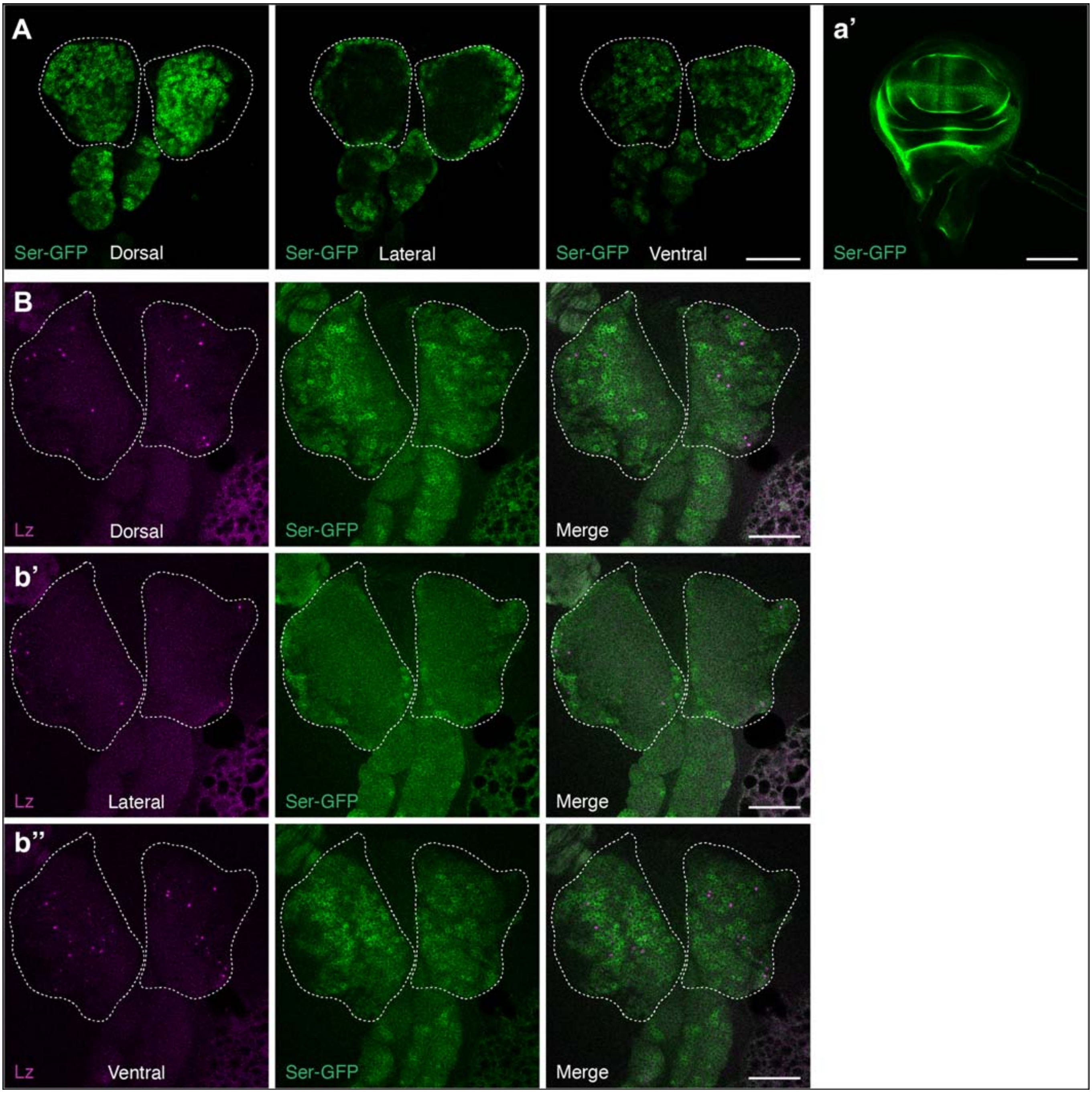

Figure EV6. Serrate expression in the larval lymph gland.

A Serrate eGFP protein fusion (Nagarkar-Jaiswal et al, 2015) appears restricted to a shallow layer of cortical plasmatocytes (**A**, **B**, **b′**, **b″**) and absent in the medullary prohaemocytes (**a′**, **b′**) of L3 larvae imaginal discs. This Ser-GFP fully recapitulates the endogenous expression in the wing disc when compared to published Serrate immunostainings (Lai, 2005) based on its pattern in the wing imaginal disc (**a′**). Crystal cell differentiation (revealed with anti-Lz) is limited to regions of Serrate expression (**B**, **b′**, **b″**). Dotted lines define the contour of the primary lymph gland lobes. Scale bars represent 100 μm.

© The Author(s)
